# Color Science in Esthetic Dentistry: Contemporary Advances in Shade Selection, Optical Properties, and Restorative Integration

**DOI:** 10.7759/cureus.116870

**Published:** 2026-09-24

**Authors:** Douglas J Silva Salas, Jennifer Herrera Pelaez, Yorilmy Pinto Guevara, Adriana Rey, Cathy Huerta Mauricio

**Affiliations:** 1 Dentistry, Universidad José Antonio Páez, San Diego, VEN; 2 Periodontics, Pontificia Universidad Javeriana, Bogota, COL; 3 Dentistry, General Practice Office, Caracas, VEN; 4 Dentistry, Universidad de San Martín de Porres, Lima, PER

**Keywords:** dental ceramics, dental esthetics, digital dentistry, optical properties, shade selection, spectrophotometry, tooth color, translucency

## Abstract

Accurate dental color reproduction is essential for natural-looking restorations and patient satisfaction. This narrative review examines the biological and optical foundations of tooth color, contemporary shade-selection methods, restorative materials, and clinical applications. Natural appearance results from interactions among light, enamel, and dentin, involving reflection, absorption, scattering, transmission, translucency, opalescence, fluorescence, surface texture, hue, value, and chroma. Although traditional visual shade selection is affected by lighting, dehydration, and operator perception, spectrophotometers, colorimeters, intraoral scanners, and cross-polarized photography provide more objective and reproducible measurements, with spectrophotometers showing the most consistent accuracy. Restorative outcomes also depend on material composition, thickness, translucency, layering, substrate color, and luting cement; ceramics, resin composites, hybrid ceramics, and zirconia differ substantially in optical behavior even when labeled with identical shades. Standardized digital workflows improve shade communication between clinicians and laboratories, and artificial intelligence-assisted systems are emerging but still require broader validation. Successful esthetic treatment therefore requires integrating objective measurement with clinical judgment, material-specific planning, finishing, and maintenance to reproduce the optical characteristics of natural teeth predictably.

## Introduction and background

Finding an accurate color match with a natural appearance remains one of the most demanding challenges in contemporary esthetic dentistry. As patient expectations for highly natural restorations increase, color fidelity has become a critical determinant of perceived treatment success: a restoration may satisfy biological, mechanical, and functional requirements yet be considered clinically unacceptable when its color does not harmonize with adjacent teeth. Accurate shade determination and reproduction are therefore essential not only for restorative integration but also for patient satisfaction and overall esthetic acceptance [1].

Dental color reproduction is a particularly complex process because tooth appearance is not a single, fixed chromatic characteristic but a multidimensional visual phenomenon conditioned by the interaction among the light source, dental tissues or restorative materials, the surrounding environment, and the observer's perception. Traditional visual shade selection is especially susceptible to subjective interpretation; several studies have shown significant inter-operator differences and low agreement in visual shade determination, reflecting the influence of perception, training, and individual color-discrimination ability [2]. Experimental evidence indicates that instruments designed for color measurement may provide greater reliability than unaided human vision under standardized conditions [3].

These limitations have favored a shift from predominantly subjective shade selection toward more objective, consistent, and reproducible methods of color assessment. However, new technology does not automatically guarantee accurate reproduction. A recent systematic review and meta-analysis found that intraoral scanners showed relatively high repeatability for shade determination, although their accuracy remained lower than that of spectrophotometers, emphasizing that precision and accuracy are not interchangeable in digital color assessment [4]. In addition, identical commercial shade designations do not necessarily correspond to identical optical appearances among restorative materials, further complicating the translation of recorded shade information into a clinically integrated restoration [5].

Current color science thus provides a conceptual basis for interpreting dental appearance as a continuous process encompassing perception, measurement, communication, information transfer, reproduction through restorative materials, and clinical verification. Emerging technologies, including artificial intelligence-based shade-selection systems, may further optimize objectivity and predictive accuracy; however, current evidence indicates that their performance continues to be influenced by illumination, measurement devices, color-space models, and algorithmic design, and broader clinical validation is still required [6]. Accordingly, this narrative review analyzes contemporary developments in dental color science by integrating the biological and optical foundations of natural tooth appearance with current shade-selection techniques, the behavior of restorative materials, and their clinical application.

The literature was identified through a narrative search of PubMed and related databases for English-language systematic reviews, meta-analyses, in vitro studies, and clinical and laboratory investigations addressing tooth color, optical properties, shade-selection methods, restorative materials, and esthetic outcomes, prioritizing contemporary evidence. Consistent with a narrative design, studies were selected for their contribution to the thematic synthesis rather than through a formal systematic protocol, and no quantitative synthesis was undertaken.

## Review

Methodology

We conducted this structured narrative review between January and September 2026. The literature search covered PubMed/MEDLINE, Scopus, Web of Science, and the Cochrane Library for records published between January 2010 and September 2026, with seminal earlier work retained where it established a concept, metric, or standard still in use, such as color-difference formulas and visual thresholds. The search combined controlled vocabulary and free-text terms across three concept blocks using Boolean operators: ("tooth color" OR "tooth colour" OR "dental color" OR shade OR translucency OR opalescence OR fluorescence OR "optical properties") AND ("shade selection" OR "shade matching" OR spectrophotometer OR colorimeter OR "intraoral scanner" OR "cross-polarized photography" OR "digital workflow" OR "artificial intelligence") AND (ceramic OR zirconia OR "lithium disilicate" OR "resin composite" OR "hybrid ceramic" OR veneer OR crown OR restoration). We hand-searched the reference lists of retrieved articles and relevant reviews for additional records.

Eligible records were peer-reviewed systematic reviews, meta-analyses, narrative reviews, in vitro and laboratory studies, and clinical and observational investigations, published in English, that addressed the optical foundations of tooth color, shade-selection methods and their accuracy or precision, the optical behavior of restorative materials, color-difference metrics, or esthetic clinical outcomes. Records were excluded when they addressed unrelated topics (for example, caries detection or purely mechanical testing without an optical outcome), when they were conference abstracts, opinion pieces, or non-English reports, or when insufficient methodological detail prevented interpretation. After removing duplicates, the authors screened records by title and abstract and assessed full texts against these criteria, resolving disagreements through discussion; 32 studies met the criteria and form the evidence base for this review. Consistent with a narrative design and to synthesize heterogeneous laboratory and clinical evidence into a clinically usable account, no formal risk-of-bias appraisal, Preferred Reporting Items for Systematic Reviews and Meta-Analyses (PRISMA) flow diagram, or quantitative synthesis was undertaken.

Literature synthesis

Fundamentals of Tooth Color and Optical Properties

Dental color perception is a complex optical phenomenon that goes beyond identifying a single shade. The appearance of natural teeth results from the interaction of light with enamel and dentin and is influenced by their composition, structure, thickness, and surface characteristics, which determine how light is absorbed, scattered, transmitted, and reflected to produce the appearance perceived by the observer [7,8]. Color is commonly characterized through three fundamental dimensions, hue, value, and chroma, describing the basic color, its lightness, and its saturation, respectively; these alone do not fully explain natural tooth appearance, and additional optical properties, particularly translucency, opalescence, and fluorescence, contribute to the vitality and natural look of dental tissues [8].

Enamel and dentin interact with light through absorption, scattering, transmission, and reflection. Dentin strongly influences the underlying hue and chroma, whereas enamel modifies the final appearance through its thickness and translucency [7,9], so variations in their relative thickness can substantially alter perceived color: the cervical region, with thicker dentin and thinner enamel, tends to appear darker, whereas the incisal region shows greater translucency owing to its greater enamel contribution [7]. Translucency, an intermediate condition between transparency and opacity, determines the extent to which light passes through dental tissues and restorative materials, and enamel and dentin exhibit different light-transmission behaviors that restorative materials should reproduce for natural optical integration [7,8]. Material composition and thickness also matter: increasing restorative thickness generally decreases light transmission and improves masking [10], and differences in filler composition and organic matrix affect resin composite translucency [11].

Opalescence results from wavelength-dependent scattering of visible light: natural enamel may appear bluish under reflected light and orange-brown under transmitted light because shorter blue wavelengths are scattered more strongly, a phenomenon to which hydroxyapatite crystals contribute and which provides optical depth, lightness, and vitality [8]. Fluorescence occurs when dental tissues absorb ultraviolet radiation and emit visible blue-white light, predominantly within the 440 to 490 nm range; dentin fluoresces more than enamel because of its organic fluorophores, structural features such as dentinal tubules and Hunter-Schreger bands may modulate intensity, and resin composites show material-dependent differences in fluorescence [8,11]. These principles have direct restorative implications because ceramic and resin-based materials differ in composition and microstructure, and therefore in optical behavior; dentin porcelain primarily influences hue and chroma, whereas enamel porcelain predominantly affects lightness and translucency [9,11,12]. Predictable esthetic integration thus requires understanding dental color as the combined result of color dimensions, tissue architecture, optical phenomena, material composition, and restorative thickness.

Quantifying these appearance characteristics requires a defined color space and a way to express color differences. Dental color is most often recorded in the Commission Internationale de l'Éclairage (CIE) Lab* (CIELAB) color space, where L* denotes lightness, a* the red-green axis, and b* the yellow-blue axis, and the total difference between two colors is expressed as ΔE [13,14]. Because CIELAB is not perceptually uniform, the CIE 2000 color-difference formula (CIEDE2000) (ΔE00), which corrects for lightness, chroma, and hue non-uniformity, more closely mirrors human judgment and is increasingly preferred for dental applications [15]. Clinically meaningful interpretation depends on visual thresholds: the widely cited 50:50% perceptibility and acceptability thresholds are ΔEab of 1.2 and 2.7 for CIELAB and ΔE00 of 0.8 and 1.8 for CIEDE2000, meaning that differences below the perceptibility threshold are generally undetectable and those below the acceptability threshold are usually judged clinically acceptable [16]. These thresholds provide the objective yardstick against which the shade-selection methods and materials discussed below should be evaluated.

Contemporary Shade-Selection Techniques

Visual shade selection has traditionally relied on subjective evaluation using standardized shade guides for comparison with the natural dentition; the guides provide a reference, but the process remains influenced by individual perception and clinical conditions. Instrumental shade selection represents an evolution toward acquiring chromatic information through devices that quantitatively record and analyze color, primarily to reduce operator-dependent perception and provide more consistent, reproducible results. Available evidence indicates that digital and instrumental methods can achieve greater accuracy and precision than purely visual selection, although performance still depends on device type and standardization of the clinical procedure [17-19].

Among instrumental methods, spectrophotometers are a principal approach because they provide objective, quantifiable measurements and, across the methods evaluated, show the highest overall accuracy and precision [4,17]. Colorimeters likewise transform chromatic perception into quantifiable information and are included among the digital and instrumental tools available for dental color analysis [17,18]. Cross-polarized photography, an evolution of conventional digital photography, improves visualization and recording of chromatic information by reducing surface reflections, facilitating assessment of internal chromatic characteristics, and improving documentation consistency [18,20]. Digital workflows integrate these technologies within a digital clinical process, allowing information from photography and instrumental devices to be stored, analyzed, compared, and communicated as a complementary record [18,19].

When comparing instrumental methods, it is important to distinguish accuracy from repeatability: a device may produce highly repeatable measurements across successive evaluations without necessarily yielding values closest to the reference, a distinction central to interpreting the performance of contemporary technologies [4,17]. Intraoral scanners illustrate this, offering strong repeatability but comparatively lower accuracy than spectrophotometers [4,21]. In visual selection, lighting conditions, dehydration, and operator variability further influence reliability, so standardized conditions, including a controlled background and illuminant, are essential when assessing and comparing methods [17,22]. Overall, instrumental methods are valuable because they reduce the influence of individual perception and yield more consistently usable quantitative information; spectrophotometers show the most consistent accuracy, precision, and reliability, whereas other instrumental and digital methods may offer advantages in reproducibility and technological integration [4,17,19]. Critically, no device is free of limitation: contact spectrophotometers with small measuring apertures sample only a limited tooth area and can misrepresent the polychromatic, translucent nature of the whole tooth, intraoral scanners trade accuracy for full-arch mapping and workflow integration, and all instruments remain sensitive to edge-loss effects, positioning, calibration, and ambient light, so instrumental readings should be interpreted against validated perceptibility and acceptability thresholds rather than treated as absolute [14,16,23].

Optical Properties of Restorative Materials

The esthetic success of a restoration depends on its ability to reproduce the complex interaction between light and natural tooth tissues. Incident light may be reflected, absorbed, scattered, or transmitted, and these interactions determine color, translucency, opacity, fluorescence, and opalescence; natural enamel is relatively translucent and primarily modifies value, whereas dentin is more opaque and largely determines hue and chroma, so restorative materials must reproduce not only the selected shade but also the optical behavior of enamel and dentin under different lighting conditions [8,10]. Dental ceramics provide excellent color stability and tooth-like optics: feldspathic ceramics are highly translucent because of their glass-rich composition, whereas increasing crystalline content increases strength and opacity, and lithium-disilicate glass-ceramics combine a translucent glass matrix with reinforcing crystals; their final appearance is nonetheless influenced by translucency level, thickness, underlying tooth color, and resin-cement shade, and systems of different composition cannot be expected to produce identical results even at the same nominal shade [11].

Resin composites contain an organic resin matrix and inorganic filler particles, and their optical behavior is governed by the refractive-index relationship between these components and by filler size, distribution, and concentration; well-matched refractive indices scatter less light and increase translucency. Contemporary systems provide enamel, dentin, body, and effect shades of different opacity, but translucency and fluorescence vary significantly among brands, so materials with the same shade designation may not be optically interchangeable [10]. Hybrid ceramics, including polymer-infiltrated ceramic networks and highly filled resin-based computer-aided design and manufacturing (CAD/CAM) materials, combine a ceramic component for optical stability and wear resistance with a polymer network for resilience and machinability; their translucency and masking depend on composition and thickness, and 1 mm hybrid CAD/CAM specimens masked dark backgrounds more effectively than 0.5 mm specimens, indicating that thickness may outweigh the manufacturer's translucency designation [9].

Zirconia was traditionally considered relatively opaque because its polycrystalline structure strongly scatters light; newer formulations with greater cubic-phase content improve translucency because cubic crystals are optically isotropic and reduce birefringent scattering, yet highly translucent zirconia remains generally less translucent and less opalescent than lithium disilicate and natural tissues, and porosity, grain size, phase composition, pigments, additives, and sintering conditions all affect light transmission [7]. Layering techniques reproduce the anatomical and optical organization of a natural tooth, with an opaque or chromatic dentin composite forming the internal body and a more translucent enamel composite modifying value and creating depth; light-transmittance studies show that changing enamel-layer thickness significantly alters the final result, so equal layers of enamel and dentin materials do not necessarily reproduce natural tissue [8], and the optimal dentin-to-enamel thickness ratio differs among ceramic systems, confirming that layering protocols should be material-specific [12]. In general, increasing thickness decreases translucency while improving masking of a dark substrate, whereas thin restorations transmit more light and are more strongly influenced by the underlying tooth, abutment, or cement; optical compatibility therefore requires coordinated selection of material composition, shade, translucency, thickness, layering strategy, substrate color, and luting cement.

Clinical Integration of Color Science

Clinical integration is where color science becomes measurable, and the FDI World Dental Federation criteria supply the vocabulary that makes esthetic performance comparable across studies, grading surface luster, staining, color match, translucency, and esthetic anatomical form on the same scale applied to functional and biological outcomes [24,25]. Ceramic veneers remain the most demanding application because thin restorations transmit rather than mask the substrate. A systematic review and meta-analysis reported high survival across feldspathic, leucite-reinforced, lithium disilicate, and zirconia veneers, with most failures attributable to fracture and debonding rather than color mismatch, indicating that optical success is generally preserved once bonding remains stable [26]. Substrate character nonetheless governs predictability, since veneers bonded to discolored dentin, composite, or endodontically treated teeth show higher complication rates than those bonded to sound enamel, and in these situations opacity selection, ceramic thickness, and try-in paste verification carry more weight than shade-tab choice [27].

For crowns, the shift toward translucent monolithic zirconia has reduced chipping while introducing a different optical problem, because higher translucency lowers masking capacity over dark preparations; pooled evidence confirms favorable long-term survival for zirconia-based restorations, with esthetic complications reported far less frequently than technical ones [28]. Direct restorations follow a different failure trajectory: anterior composites show acceptable longevity but fail predominantly for esthetic reasons, with marginal discoloration, surface staining, and loss of luster driving replacement long before fracture [29], so finishing, polishing, and material selection become decisive, and aging data confirm that contemporary resin-based materials differ substantially in how their color and translucency drift after exposure to staining media [30].

Communication with the laboratory is the step where documented color is most often lost. Objective workflows that combine standardized photography with numeric shade quantification allow color to be transferred as coordinate data rather than a subjective tab name, improving reproduction and letting the technician verify the result before delivery [31]; systematic evaluation confirms that instrumental and photographic methods outperform visual selection in repeatability, although accuracy still depends on calibration, illumination, and operator training [18]. Digital smile design extends this logic from single restorations to full rehabilitation, integrating facial reference, dental proportion, and functional parameters into one planning environment, and recent meta-analytic evidence indicates that artificial intelligence-assisted smile design significantly improves patient and clinician satisfaction and esthetic outcomes, although heterogeneity remains high and prospective confirmation is required [32]. Color stability over time completes the clinical picture: maintenance protocols, polishing recall, and realistic expectations regarding staining should be established during treatment planning rather than after complaints arise, and recording baseline esthetic status with FDI criteria allows objective documentation of change and distinguishes true material degradation from extrinsic staining that responds to simple repolishing [24,25,30].

Current Challenges and Future Directions

Despite clear progress, several challenges persist. No single method yet combines the accuracy of spectrophotometry with whole-tooth imaging and the workflow integration of intraoral scanners, and instrument performance still depends on illumination, calibration, background, and operator standardization [4,17,22]. Standardization is a recurring gap: shade-guide systems and color-space models are not fully harmonized, identically labeled materials differ optically between manufacturers, and reported outcomes are difficult to compare without common conditions and metrics [5,18]. Artificial intelligence-based shade matching and digital smile design are promising for objectivity and patient-centered planning, but current evidence is heterogeneous and often lacks prospective clinical validation [6,32]. A related problem is interpretation: many studies report mean color differences without referencing them to perceptibility and acceptability thresholds, so statistically significant differences may be clinically irrelevant and vice versa; consistent use of CIEDE2000 with the accepted 50:50% thresholds would make results comparable and clinically meaningful [15,16]. Future research should prioritize harmonized measurement protocols and color spaces, whole-tooth and multi-region shade mapping, validated prediction of final restorative appearance from recorded data across material systems, and longitudinal assessment of color stability against threshold-based criteria. For daily practice, the evidence supports using an objective device, preferably a spectrophotometer, under standardized conditions as an adjunct to trained visual assessment, selecting materials by their measured optical behavior rather than nominal shade alone, and transferring color to the laboratory as quantified data with photographic support.

## Conclusions

Natural tooth color is a multidimensional optical phenomenon produced by the interaction of light with enamel and dentin and shaped by hue, value, chroma, translucency, opalescence, fluorescence, surface texture, and gloss. Reproducing it predictably requires more than matching a shade tab. Objective measurement, particularly spectrophotometry supported by cross-polarized photography and digital workflows, reduces the subjectivity of visual selection, while restorative success additionally depends on material composition, thickness, translucency, layering, substrate color, and luting cement, since ceramics, composites, hybrid ceramics, and zirconia behave differently even at the same nominal shade. Successful esthetic treatment therefore integrates objective measurement with clinical judgment, material-specific planning, meticulous finishing, standardized clinician-laboratory communication, and long-term maintenance. As instrumental and artificial intelligence-assisted methods mature and become better standardized and validated, they are likely to make esthetic outcomes increasingly predictable and patient-centered.
